# Comparative Evaluation of the Intratubular Penetration Ability of Two Retrograde Obturation Techniques in Micro-Endodontic Surgical Procedure: An In Vitro Study with Confocal Laser Scanning Microscopy

**DOI:** 10.3390/dj13110509

**Published:** 2025-11-03

**Authors:** Alberto Casino Alegre, Michell Ramírez López, Manuel Monterde Hernández, Susana Aranda Verdú, Jorge Rubio Climent, Antonio Pallarés Sabater

**Affiliations:** 1 Department of Endodontics and Restorative Dentistry, School of Medicine and Dentistry, Catholic University of Valencia, 46001 Valencia, Spain; michellsayo@gmail.com (M.R.L.); manuel.monterde@ucv.es (M.M.H.); susana.aranda.verdu@gmail.com (S.A.V.); jorrucli@hotmail.com (J.R.C.); antonio.pallares@ucv.es (A.P.S.); 2 Doctoral School, Catholic University of Valencia, 46001 Valencia, Spain

**Keywords:** apical surgery, apicoectomy, CLSM, conventional technique, lid technique, micro-endodontic surgery, retrograde obturation

## Abstract

**Background:** The development of calcium silicate materials and new techniques have resulted in significant clinical benefits in endodontics and microapical surgery. The objective of this investigation was to analyze the percentage of dentinal tubule penetration of two retrograde obturation techniques in microapical surgery, namely the conventional technique and the lid technique. **Methods:** 60 single-root human teeth were selected, which were divided into two groups *(n* = 30). These teeth were subjected to an endodontic procedure using the single-cone technique. They were prepared with apicoectomy and 3 mm apical retrocavity and then obturated using two retrograde obturation techniques with bioceramic materials: TotalFill RRM fast set Putty^®^ (RRM) using the conventional technique and TotalFill BC Sealer HiFlow^®^ (HiFlow) and RRM using the lid technique. The teeth were selected and evaluated using 1 mm portions in the apical third. In each case, the images were obtained using a *Leica TCS SP8 Confocal Microscope* (CLSM). The extent of penetration into the dentinal tubule regions was measured using AutoCad^®^. **Results:** Statistical analyses were performed using the Levene test (*p* ≤ 0.05) and Student’s *t*-test (*p* ≤ 0.05). Analysis of the penetration area of calcium silicate materials into the dentinal tubules revealed that the relative penetration percentages were higher when using the conventional technique with the RRM than the lid technique with RRM + HiFlow in the apical third evaluated. **Conclusion:** The conventional technique yields significantly better outcomes, showing statistically significant differences in the percentage of penetration into the intratubular area compared to the lid technique.

## 1. Introduction

The primary objective of root canal treatment is to reduce the bacterial load within the infected canal system of a tooth, thereby preventing the possible infection and facilitating the healing of pulpal and periapical tissues. Ultimately, this process aims to preserve a fully functional tooth [1]. In cases of root canal treatment failure, nonsurgical retreatment is typically the first-line approach [1]. However, if retreatment proves ineffective or has an uncertain prognosis, endodontic microsurgery becomes a vital intervention, and often the final option to preserve the tooth [2,3].

Retrograde obturation is one of the most critical steps in endodontic microsurgery. Among available materials, bioceramics are considered the gold standard for the retrograde filling [4,5].

Bioceramics were introduced into endodontics in the early 1990s as a novel and useful group of dental materials. Subsequently, an extensive review of research into these materials was published [6]. They are biocompatible ceramic materials composed of metal oxides, characterized by enhanced sealing ability. Furthermore, their antibacterial and antifungal properties have lead to widespread use in both medicine and dentistry [7]. They are also able to promote reabsorption and regeneration in human tissue. These materials include reabsorbable alumina and ceramic glasses, calcium phosphate, zirconia, calcium silicates, bioactive glasses and hydroxyapatite [7,8].

In general, bioceramic materials are bioactive, with calcium-silicate-based sealer being the most commonly used due to their favorable physical and chemical properties. This is especially relevant in endodontics, where both biocompatibility and bioactivity are essential [9,10].

Endosequence BC Sealer^®^ (Brasseler, Savannah, GA, USA) contains calcium phosphate, which facilitates the formation of hydroxyapatite during setting and promotes bonding between dentinal and filling materials, thereby increasing the strength and durability of the teeth. Endosequence BC HiFlow^®^ also exhibits additional properties; its fluidity increases when exposed to heat, which may enhance its ability to penetrate dentinal tubules [11,12,13].

Endosequence^®^ and Endosequence HiFlow^®^ share the same composition as TotalFill^®^ (FKG Dentaire SA, La-Chaux-de-fonds, Switzerland) and HiFlow [14]. The composition of HiFlow includes zirconium oxide, tricalcium silicate, dicalcium silicate, calcium hydroxide and radiopacifying agents [15]. RRM (FKG Dentaire SA, La Chaux-de-Fonds, Switzerland) is a relatively new bioceramic material [16]. According to the manufacturer, its composition includes zirconium oxide, tantalum pentoxide, monobasic calcium phosphate, calcium silicates and bulking agents [16]. This pre-mixed, ready-to-use material is available in putty consistency, presented either in a jar or a syringe [16]. The syringe presentation has a shorter setting time of approximately 20 min [17]. Endosequence^®^ and TotalFill^®^ share a common setting reaction in which powdered calcium silicates form a gel composed of calcium silicate and calcium hydroxide upon hydration. Water subsequently reacts with the calcium silicates, leading to the precipitation of calcium silicate-hydrate, which ultimately results in the formation of hydroxyapatite [18]. Moisture plays a key role in this process by regulating the degree of hydration and setting of the material [18,19].

Regarding techniques, the conventional obturation method uses putty-consistency material, which is compacted in successive layers within the retrograde preparation [4]. A significant advantage is the ability to compact the material using specific instruments, thereby creating a retrograde filling without voids. However, placing the putty material in deep retrograde preparations can be challenging with this technique. To address this limitation, a new retrograde filling approach, known as the lid technique, was introduced by Nasseh et al. [2]. This technique combines two bioceramic consistencies, fluid and putty. However, the compaction is more challenging than in the conventional technique, as the sealer tends to overflow during retrograde preparation; we may consider this an important disadvantage due to the condensation of the material putty. A key advantage of this technique is the ease of delivering the fluid material into deep retrograde preparations (6–9 mm) because the specific point allows to reach this depth in an adequate way. Retrocavity preparation and obturation have been shown to improve the success rates of apical microsurgery [4]. The development of bioceramic materials has resulted in huge benefits in clinical practice use, such as sealing obturation materials [1,20].

Although the literature extensively documents the ability of bioceramic sealers to penetrate dentinal tubules through various obturation techniques and analytical methodologies such as confocal microscopy and standardized 1 mm thick sections [15,21], there is a notable lack of studies directly comparing the conventional retrograde obturation technique with the lid technique. One study evaluated void volumes and the effect of blood contamination on the surface microhardness of a calcium-silicate-based repair cement, a sealer, and their combination (lid technique) [22], but did not assess the intratubular penetration ability of the materials. The lid technique, which combines flowable and putty consistencies [22], has been scarcely described in the literature [2], and its effectiveness regarding dentinal tubule penetration remains largely unexplored. In this context, the present study was designed to address this knowledge gap by directly comparing both techniques under standardized conditions.

The null hypothesis (H_0_) posits that there are no significant differences in the area of penetration into the dentinal tubules between the conventional and lid technique. The overall aim of this comparative study is to evaluate two retrograde obturation techniques in terms of dentinal tubule penetration and to assess their influence on apical sealing.

## 2. Materials and Methods

This study was approved by the UCV Research Ethics Committee, (code UCV/2019–2020/001).

### 2.1. Sample Selection

A sample size of 30 specimens per group was chosen to ensure adequate statistical power and minimize biological variability.

A total of 60 single-rooted teeth, comprising canines, premolars, and incisors, were selected for this study. All teeth had been extracted due to periodontal disease. Teeth presenting severe curvatures, immature roots, resorption, previous root canal treatment, canal obliteration, or fractures were excluded. The selected teeth were soaked in sodium hypochlorite (NaOCl) (Veltech Associates, Philadelphia, PA, USA) for one hour for disinfection. Subsequently, calculus and residual periodontal ligament were removed from the root surfaces using a universal 7–8 curette (Bader^®^, Vigo, Spain) and the teeth were stored in saline solution [15].

### 2.2. Preparation of the Root Canal

Two parallel radiographs were taken in buccolingual and mesiodistal orientations for each tooth, from different angles, to verify the existence of a unique canal. Following the access of the canal with a conical bur (Intensiv, Montagnola, Switerzland) and high speed irrigation, the canal was identified using a specific instrument DG16 (Bader^®^, Vigo, Spain). The crown of the tooth was sectioned at the cemento-enamel junction with a disc handpiece while using refrigeration with water. A 10 K manual file was then placed into the canal, and the working length (WL) was determined to be 0.5 mm from the apical constriction through visual inspection. Protaper Gold^®^ (Dentsply Sirona, Ballaigues, Switzerland) was used to shape all root canals according to the manufacturer’s guidelines [20].

S1 and S2 files were used for root shaping, with brushing and circumferential movements at working length up and down and in and out, whereas finishing files F1 and F2 were used with a pecking movement using a Gold Reciproc^TM^ motor (VDW, Münich, Germany). The canal was irrigated with a 5.25% sodium hypochlorite after the use of each file. The patency was confirmed by passing a manual K-file with small diameter through the apical constriction once the preparation process was finished [15].

For the final irrigation, the canals were treated with hypochlorite for 60 s, followed by 17% EDTA (Dentaflux, Madrid, Spain) for 60 s. A final rinse was conducted using saline solution, which was also utilized between the different irrigants (NaOCl, EDTA, and chitosan (Arnandis Pharmacy, Valence, Spain). The irrigants were activated with the EDDY^®^ sonic tip (VDW, Munich, Germany) using an Air Scaler. The canals were dried using F2 paper points. This mechanochemical sample protocol method was consistently applied in all samples [15].

### 2.3. Obturation of Root Canals

A single-cone of gutta-percha ProTaper Gold^®^ Conform Fit^TM^ F2 and AH Plus^®^ sealer (Dentsply Sirona, Ballaigues, Switzerland) was used to seal the canal correctly.

After sealing the samples, they were kept in a laboratory incubator at 37 °C and complete humidity for 7 days, ensuring a perfect fit of the materials. No changes were observed after the insertion of the materials nor after the setting.

### 2.4. Apicoectomy of the Samples

After this seven-day period, the teeth were washed with physiological saline solution. The 3 mm cut was performed in the apical sector of the roots using a lance diamond bur (Komet, Lemgo, Germany) and at a high speed at an angle of 90°. The perpendicular cut was checked using a graduator.

### 2.5. Retrocavity

Once the cut had been made, a 3 mm section was prepared using an s12/70D (diamond-coated 30 µm, 3 mm, 9% taper) ultrasonic tip (Acteon-Satelec, Merignac, France) with ultrasonic D600 led (DTE) compatible with ultrasonic Acteon-Satelec tips, which was inserted to the whole length of the active tip (3 mm). The cavity was standardized using an ultrasonic tip with a consistent diameter, and the same type of tip was used for all samples.

### 2.6. Retrograde Obturation of the Samples

The cements were prepared according to the manufacturer’s instructions.

Prior to application of the bioceramic cements, the root was carefully prepared with the ultrasonic tip until 3 mm with water cooling and saline solution. The dentinal surface was conditioned with a chitosan–hydroxyapatite precursor for 30 s. Hashmi et al. [23] observed that the chitosan–hydroxyapatite precursor enhances dentin surface wettability to facilitate greater bioceramic sealer penetration in the dentin. The retropreparation was then dried with paper points F2 (Dentsply Sirona, Ballaigues, Switzerland).

### 2.7. Study Groups

**Figure 1:**
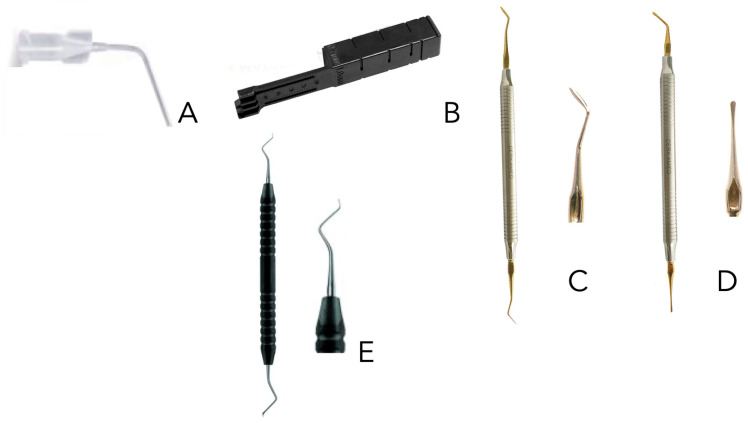
Instruments used in the obturation techniques. (**A**): specific point to the sealer; (**B**): Matrix MTA+ to create the cones; (**C**): MX1 spatula; (**D**): MX3 spatula; (**E**): Condenser.

Group 1 (*n* = 30): Lid technique (HiFlow + RRM). In this case, the lid technique was applied. The cap was removed from the TotalFill BC Sealer HiFlow syringe, a BC tip (Figure 1A) was firmly attached by turning the syringe cone once clockwise. 0.1% Rhodamine BTM (Sigma-Aldrich corp., St. Louis, MO, USA) was added by weight to the bioceramic sealer. This combination was then mixed carefully until a pale pink mixture was obtained; this mixture was placed in the syringe. The syringe tip was inserted, 3 mm, into the apical area, and the cavity was filled with HiFlow until a slight overfilling of the sealer was observed. The RRM was mixed with 0.1% rhodamine B by weight, and then put in slot of the Matrix MTA+ (Figure 1B) (Cerkamed, Stalowa Wola, Poland) to create a specific cone. This small conic form of RRM was applied with the spatula MX1 and MX3 (Figure 1C,D) (Cerkamed, Stalowa Wola, Poland) in the retropreparation and it was condensed using the condenser (Figure 1E) (Ref: AESC-DE445R Sanhigia^®^, Saragossa, Spain). The consistency was checked until it could not be removed with a wet microbrush using gentle horizontal movements.

Group 2 (*n* = 30): conventional technique (RRM). Thus, the cap was removed from the syringe and a small portion of the material was removed; 0.1% rhodamine B, by weight, was added to the calcium-silicate-based cement. This combination was then mixed carefully to obtain a pale pink mixture. The RRM was put in slot of the Matrix MTA+ (Figure 1B) (Cerkamed, Stalowa Wola, Poland) to create a specific cone. This small conic form of RRM was applied with the MX1 and MX3 spatula (Figure 1C,D) (Cerkamed, Stalowa Wola, Poland) in the retropreparation and it was condensate with the condenser (Figure 1E) (Ref: AESC-DE445R Sanhigia^®^, Saragossa, Spain). The material was condensed inside the preparation from top to bottom to prevent air from becoming trapped inside, until the preparation became completely sealed. Excess material was then removed using cotton swabs.

All samples were stored at 100% relative humidity and 37 °C in a laboratory incubator to ensure proper setting of the bioceramic materials.

### 2.8. Sample Cutting

**Figure 2:**
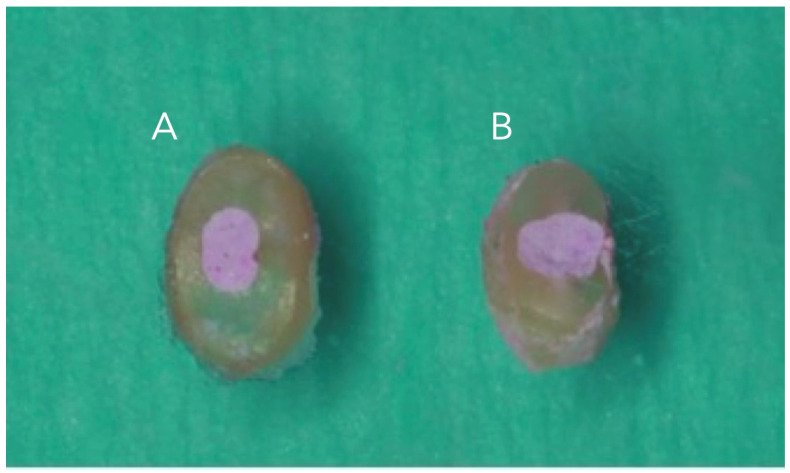
Representative image 1 mm cuts slices, (**A**): lid technique and (**B**): conventional technique.

The root was cut into one apical part (the apical third was taken by measuring a length of 2 mm) and horizontal cuts were made using a 0.3 mm diamond disc handpiece (Komet, Lemgo, Germany) with water refrigeration. Slices with a thickness of 1 mm were then obtained and polished with Sof Lex (3M ESPE (TM TM) St. Paul, MN, USA) discs. The slices were mounted on a slide using Super Glue-3 (Loctite, Valence, Spain) (Figure 2).

### 2.9. Imagen Analysis: Confocal Laser Scanning Microscopy (CLSM)

**Figure 3:**
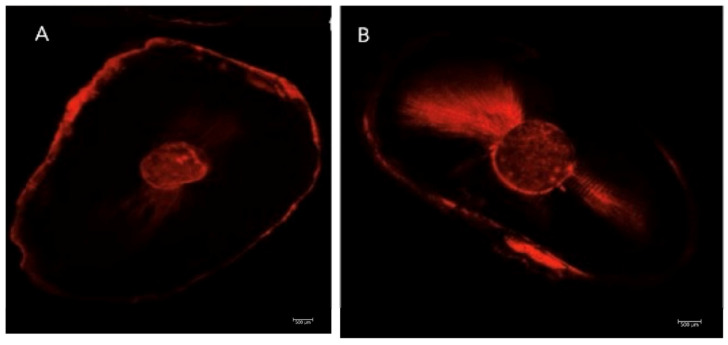
Representative image, scale 500 µm^2^; (**A**): lid technique and (**B**): conventional technique.

Images were obtained using a CLSM (Leica TCS SP8 Confocal Microscope (Leica Microsystems GmbH, Wetzlar, Germany) at the University of Valencia (Central Service for Experimental Research). Sample analysis was performed at a 5× magnification. All images were saved and subsequently analyzed (Figure 3).

Initially, each image was scaled to 500 µm to ensure accurate measurement of all elements. AutoCAD^®^ software (AutoCAD 2025) was used to measure the percentage of intratubular penetration of bioceramic materials, the images were obtained and saved in a technical file.

**Figure 4:**
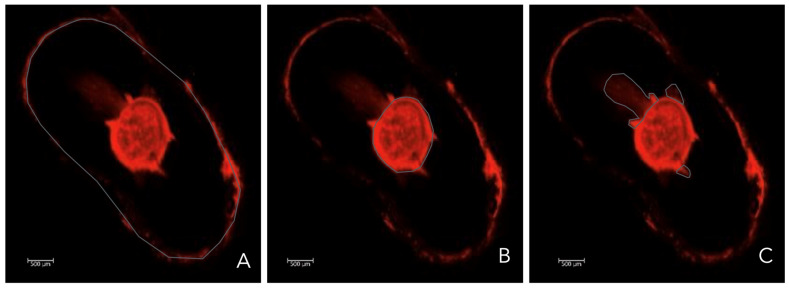
Representative image, the gray line represented; (**A**): Total area of the sample. (**B**): Area of the canal (**C**): Area with dentinal penetration of the bioceramic sealer.

The specific AutoCad^®^ function was applied to measure the percentage intratubular penetration area. It added the area of the canal (Figure 4A) and area with dentinal penetration of the bioceramic material (Figure 4B) divided by the total area of the sample (Figure 4C) and multiplied (×100).

**Figure 5:**
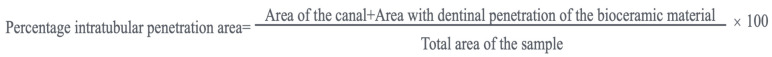
Formulae to calculate the percentage intratubular penetration area.

This value represented the percentage of intratubular penetration area of the bioceramic material (Figure 5).

### 2.10. Statistical Analysis

Statistical analysis of the data was conducted using the SPSS 23 software suite with a confidence level of 95% and by considering those results with a comparative *p*-value of less than 0.05 (*p* ≤ 0.05) to be statistically significant. Levene test and *t*-test were used to analyze the study data.

## 3. Results

### Study or the Percentage Intratubular Penetration Area for the Bioceramic Cements in the Dentinal Tubules

The following penetration percentages were obtained using the two techniques. The penetration index for the lid technique was 38.69 ± 12.01% and that of the conventional technique was 74.25 ± 23.14% (Table 1).

It can be seen that the penetration index for the lid technique was lower than that of the conventional technique. Subsequently, we conducted the corresponding Levene equality of variance test.

The *p*-value for the Levene test was 0.236, which is higher than 0.05; therefore, we assumed that the variances for the two samples were equal. Therefore, we performed a comparative t-test assuming equal variances.

The *p*-value for the *t*-test was 0.007, which is less than 0.05. We found evidence to show that the percentage of intratubular penetration areas for the two techniques are significantly different, the area for the conventional technique being significantly higher than the lid technique.

## 4. Discussion

The main objective of micro-endodontic surgery is to achieve a hermetic seal of the retrograde cavity, thereby preventing potential reinfection. The preparation and sealing of the retrograde cavity have been shown to significantly enhance treatment success rates [4]. The development of bioceramic materials has enabled the incorporation of these novel materials into micro-surgical procedures, highlighting the need to study their proper handling and to select the appropriate consistency for each clinical case, with the aim of enhancing treatment outcomes.

The tubular penetration area can serve as an indicator of the sealing ability of endodontic sealers to the walls of the root canal. To allow visualization of the sealer within the dentinal tubules using CLSM in in vitro studies, staining of the material is required.

The CLSM is an effective tool for the magnification and evaluation of samples that has been used in previous studies for this purpose [15,24]. Although a scanning electron microscope (SEM) is a viable alternative, we ruled it out in this study due to the fact that SEM samples need different manipulations in order to be visible. This specific manipulation may be responsible for the production of artifacts and compromise accurate visualization of the samples [25].

Rhodamine B is a fluorescent dye commonly used to evaluate dentinal penetration of bioceramic materials, as the small amount used (0.1% by the way of the sealer or RRM) does not alter the material’s qualities [26]. In our study, we used rhodamine B mix HiFlow and RRM with the aim of visualizing them in the different techniques used. Rhodamine B would not affect penetration in our study as this may be one of the most suitable methods of working in vitro when studying intratubular penetration.

In the present study, the percentage of intratubular penetration was quantified using an area-based approach that considers both the root canal lumen and the adjacent dentinal regions exhibiting sealer penetration, relative to the total cross-sectional area of the specimen. This approach aligns with that employed by Gülmez et al. [21], who also used area-based measurements under CLSM to quantify the extent of sealer infiltration into dentinal tubules. This methodology was selected to provide a comprehensive assessment of material distribution, particularly in cases where sealer penetration is uneven along the canal walls, as observed in specimens exhibiting the butterfly effect. By incorporating both the canal space and the surrounding penetrated dentin in the numerator, this formula captures the overall interaction of the bioceramic material with the dentinal substrate, offering a more integrated and spatially representative interpretation of sealing effectiveness.

Calcium-silicate-based cements can be a material of choice for retrograde cavity filling in apical microsurgery. RRM and MTA have been extensively studied demonstrating superior bioactivity, antimicrobial effects, cytocompatibility, sealing ability and biocompatibility [27]. MTA presents several weak points such as long setting time and difficulty in the manipulation and discoloration. Recently, several calcium-silicate-based cements have appeared, aimed at overcoming shortcuts of MTA [27]. Various studies have confirmed that RRM are also biocompatible materials with good sealing capacity which is suitable for retrograde filling [28,29,30]. An alternative to these materials are IRM and super EBA; they showed higher sealing ability and adequate manipulation but poor bioactivity, biocompatibility and cytocompatibility in comparison with RRM and MTA. MTA has superior cell viability to IRM [31]. In addition, the lid technique could not be used with IRM and super EBA because we would be using two different types of material and may interfere in the properties of each one.

The outcomes of this study showed that the conventional technique resulted in greater dentinal tubule penetration than the lid technique. These findings may be due to the pressure in the compaction of the material in retropreparation. This pressure applied might be different in the lid technique because of the presence of fluid sealer in the cavity. The combination of two different materials in the lid technique may be responsible for the results, as it is very difficult to compact the liquid into the cavity, often requiring the addition of more bioceramic material in putty consistency.

We acknowledge that the relatively large standard deviations observed in our results may reflect the inherent biological variability of the specimens, such as differences in dentinal tubule density, diameter, and distribution across individual teeth. Although we followed strict inclusion criteria and standardization, anatomical differences among samples, such as root canal morphology and tubule orientation, likely contributed to the variability in sealer penetration.

Additionally, the results may be attributed to the existence of a layer referred to as the “mineral infiltration zone” (MIZ) [32]. This layer might be linked to alterations in the intratubular microstructure, which could in turn alter the properties of the interfacial dentin [32].

The MIZ may result from a two-fold effect of the calcium-hydroxide-releasing cement: initially, an alkaline caustic etching, followed by the diffusion of minerals [32]. The creation of an interfacial layer between dentin and calcium-silicate-based cements has been previously reported [33,34,35]. These studies reported that the layer forms within the dentin tubules and the interfacial region of both substrates, resulting from hydroxyapatite formation. Additionally, Atmeh et al. [32] demonstrated that the MIZ develops within the intertubular structure of the dentin and is enriched with carbonate ions.

The findings of our study are similar to those reported by Arvelaiz et al. [16] using the conventional retrograde obturation technique. These authors investigated the bioceramic materials MTA and RRM and the ability thereof to prevent the microfiltration of *Enterococcus faecalis* over time. These authors concluded that both the MTA and RRM are equally suitable for use as retrograde obturation materials. We propose two principal effects in the dentinal tubules’ penetration of the bioceramic materials. One of them is the burial effect of the microorganisms inside the canal that may inactivate them. The second is the bactericide effect by contact of the material with the microorganisms.

In the investigations by Chen et al. [36], Eren et al. [37] and Donnermeyer et al. [38], the endodontic sealers Endosequence and TotalFill BC Sealer HiFlow^®^ exhibited chemical properties characterized by lower viscosity and higher flowability. The application of warm obturation techniques did not lead to any substantial chemical changes at any temperature in these materials. This finding suggest that TotalFill BC Sealer HiFlow^®^ is suitable for both thermoplastic and cold obturation techniques, although it demonstrated greater intratubular penetration when used with warm obturation techniques [15].

A comparison of our findings with those from previous studies suggests that, depending on the viscosity and fluidity, bioceramics penetrate dentinal tubules better than others [15]. RRM material is a bioceramic material with different applications. The conventional technique may enhance penetration of the bioceramic as a result of compaction of the material [39]. The chemical properties and setting time of this bioceramic (RRM) are likely to have influenced, in our opinion, its higher penetration compared to the lid technique with RRM and HiFlow, which is more fluid and has a slower setting time. The lid technique used with both bioceramic materials presented lower compaction as it was performed using a syringe to the sealer followed by placement of a putty cap [2]. The interaction and limited compaction of the materials may have affected the low penetration of the lid technique.

In terms of the clinical implications of our study, it is important to have different techniques available with various materials to address a wide range of clinical scenarios. Obturation of a 3 mm retrocavity is relatively straightforward using the conventional technique; however, in cases with a 6 mm retrocavity, achieving a proper seal without voids can be considerably more challenging. In such cases, the lid technique can be utilized effectively, providing adequate results without compromising the prognosis of the case. Although our results showed greater dentinal tubule penetration with the conventional technique, the lid technique could represent a valuable alternative in situations involving larger retrocavities.

Regarding the limitations of this investigation, the most suitable type of material for use in retrograde obturation remains uncertain, although RRM appears to be a good option based on the predictable outcomes observed in this study. There is limited information available concerning the lid technique in retrograde obturations, as most existing literature focuses on clinical cases rather than in vitro studies. We did not find similar recent studies evaluating intratubular penetration with which to directly compare our results. Therefore, further research is warranted to investigate the behavior of these new materials and, in particular, the influence of the technique used for each. It should also be noted that the results obtained from this in vitro study may differ from those observed in vivo, due to the biological variability and the more complex setting behavior of bioceramic materials in clinical environments.

## 5. Conclusions

Within the limitations of this in vitro study, the conventional retrograde obturation technique using RRM demonstrated significantly greater dentinal tubule penetration compared to the lid technique combining RRM and HiFlow. This difference is likely attributable to material consistency and compaction pressure. While the lid technique may offer advantages in cases involving deeper retrocavities, its lower penetration warrants further investigation through in vivo studies.

## Figures and Tables

**Table 1:** Descriptive statistics: N (sample size); % (percentage of dentinal penetration, shown as mean ± standard deviation); 95% confidence intervals (CIs) are also presented as percentages.

Groups	N	%	95% CI	
			Lower Limit	Upper Limit
Lid technique	30	38.69 ± 12.01	26.68	50.71
Conventional technique	30	74.25 ± 23.14	51.12	97.39

## Data Availability

The data that supports this study are available from the Catholic University of Valencia. Restrictions apply to the availability of these data, which were used under license for this study.
